# Novel Framework Based on HOSVD for Ski Goggles Defect Detection and Classification

**DOI:** 10.3390/s19245538

**Published:** 2019-12-14

**Authors:** Ngoc Tuyen Le, Jing-Wein Wang, Chou-Chen Wang, Tu N. Nguyen

**Affiliations:** 1 Institute of Photonics Engineering, National Kaohsiung University of Science and Technology, Kaohsiung 80778, Taiwan; tuyennl75@gmail.com; 2 Department of Electronic Engineering, I-Shou University, Kaohsiung 84001, Taiwan; chchwang@isu.edu.tw; 3 Department of Computer Science, Purdue University Fort Wayne, IN 46805, USA.; nguyentu@purdue.edu

**Keywords:** ski goggles lens, HOSVD, automatic optical inspection, adaptive energy analysis, parallel projection in opposite directions

## Abstract

No matter your experience level or budget, there is a great ski goggle waiting to be found.Goggles are an essential part of skiing or snowboarding gear to protect your eyes from harsh environmental elements and injury. In the ski goggles manufacturing industry, defects, especially on the lens surface, are unavoidable. However, defect detection and classification by visual inspection in the manufacturing process is very difficult. To overcome this problem, a novel framework based on machine vision is presented, named as the ski goggles lens defect detection, with five high-resolution cameras and custom-made lighting field to achieve a high-quality ski goggles lens image. Next, the defects on the lens of ski goggles are detected by using parallel projection in opposite directions based on adaptive energy analysis. Before being put into the classification system, the defect images are enhanced by an adaptive method based on the high-order singular value decomposition (HOSVD). Finally, dust and five types of defect images are classified into six types, i.e., dust, spotlight (type 1, type 2, type 3), string, and watermark, by using the developed classification algorithm. The defect detection and classification results of the ski goggles lens are compared to the standard quality of the manufacturer. Experiments using 120 ski goggles lens samples collected from the largest manufacturer in Taiwan are conducted to validate the performance of the proposed framework. The accurate defect detection rate is 100% and the classification accuracy rate is 99.3%, while the total running time is short. The results demonstrate that the proposed method is sound and useful for ski goggles lens inspection in industries.

## 1. Introduction

Ski goggles, or snow goggles, are essential for winter sporting activities like skiing and snowboarding. Factors such as an increase in disposable income and participation in outdoor recreational activities will contribute to the growth of the ski goggles market in the coming years. Skiing and snowboarding are sports that require precaution to help keep you safe as. when you cannot see well, you are more likely to injure yourself. Goggles are an essential part of skiing or snowboarding gear to protect your eyes from harsh environmental elements and injury. These sports expose your eyes to prolonged periods of harsh wind and bright sunlight. Unlike sunglasses, goggles seal your eyes from cold air, and many goggles come with lenses that block UV light, and reduce glare effects from the snow. Goggles protect your eyes from airborne snow and debris and shield them from hazards such as tree limbs and fallen branches. Ski goggles also stay securely on your head at times when sunglasses would fly off. 

Ski goggles have many components, of which the most important component is lenses, which all have an important role in improving your vision, protecting your eyes, and keeping you comfortable. Nowadays, the global ski goggles market is expected to grow at a compound annual rate. In the ski goggles manufacturing industry, defects, especially on the lens surface, are unavoidable. So far, experts have classified the defect types manually. If we know the exact type of the given defect, not only can we remove the defects but also find what part of the fabrication process has problems. Because of the importance of the ski goggles lens, the defect detection and classification process, in order to ensure the high standard of the ski goggles lens, plays an extremely important role for manufacturers.

**Figure 1:**
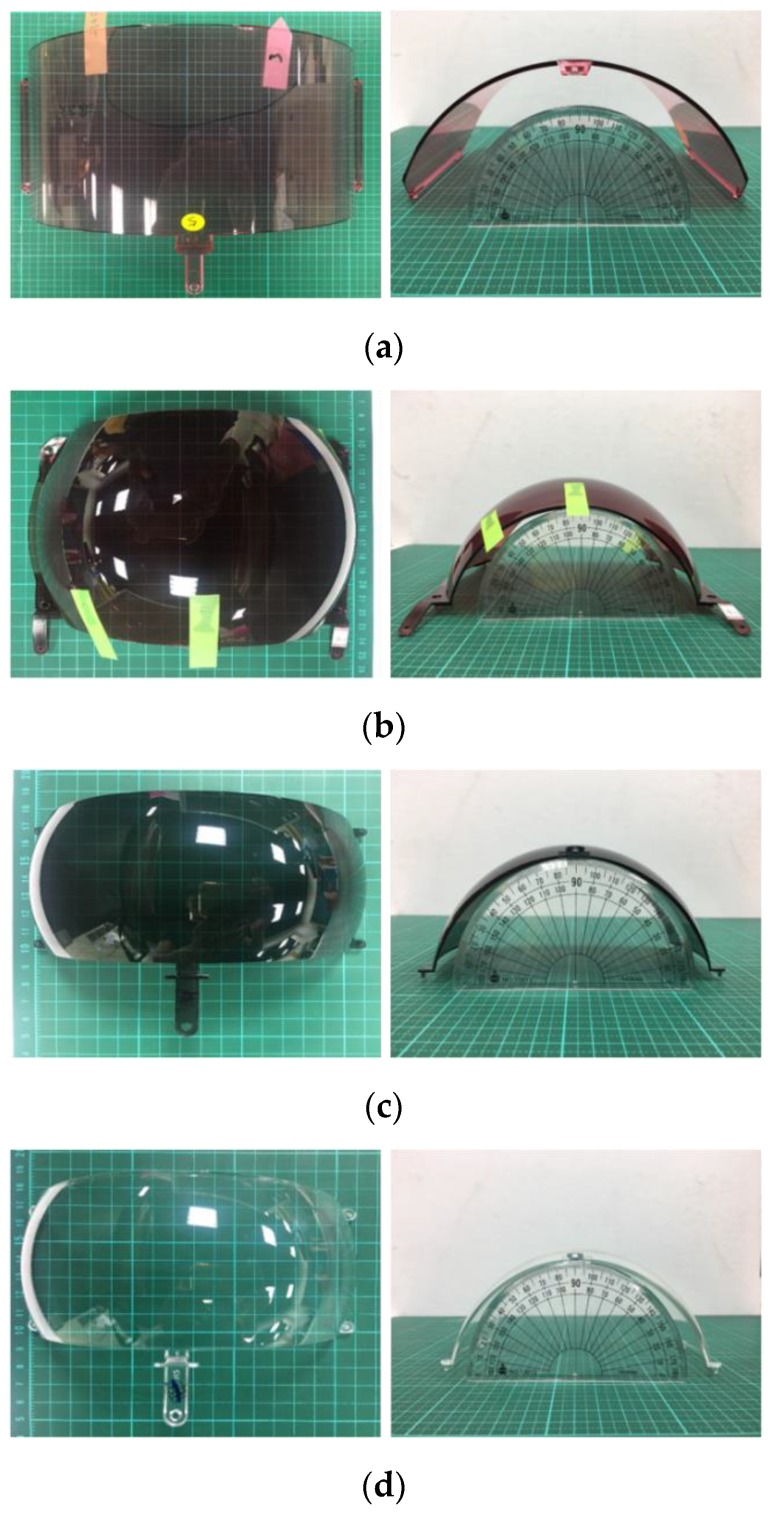
Ski goggles lens samples. (**a**) Outer rose color lens sized 205 mm × 126 mm; (**b**) outer dark red color lens sized 185 mm × 134 mm; (**c**) outer dark blue color lens sized 70 mm × 155 mm; (**d**) inner lens sized 70 mm × 155 mm.

On most goggles, the lens consists of two separate lenses, called outer lens and inner lens, as shown in Figure 1. Lenses are normally made from a polycarbonate or Trivex plastic, which makes them more impact resistant and less likely to shatter than glass or normal plastics. Virtually all ski goggle lenses today protect you 100% from UV light and prevent harmful UVA, UVB, and UVC rays entering your eyes. There are two different shapes of goggles lenses: flat and spherical. Flat lenses are flat vertically with a curve sideways across the goggles. Spherical lenses are curved in all directions and are shaped as if they were cut from the part of a sphere. The aspherical lenses are more expensive but offer better peripheral vision and have less optical distortion. 

Additionally, ski goggles lenses can be coated tints to suit different weather conditions. Yellow, rose-tinted lenses are best for a snowy conditions; orange, gold, blue, gray tinted lenses are best for sunny conditions; and amber lenses are best for low to medium light conditions. Another solution to harsh weather conditions is to have a photochromic lens, or polarized lenses. These ski goggles are extremely useful in improving vision, but also make the defect inspection process by automatic optical inspection (AOI) system more difficult. Many goggles have a reflective coating on the lens surface that creates a mirrored effect. The tint-coated, anti-UV, or polarized lenses make the image acquisition process more difficult because they have different light reflections.

In Industry 4.0, AOI is a key machine vision-based technique used in the manufacture and test of products. AOI is an essential tool using optics to capture images of an object under test in an integrated test strategy that ensures costs are kept at the lowest possible by enabling fast and accurate fault-detection in the early period of the production chain. Recently, AOI has been increasingly used for automatic defect detection in several industrial fields, such as printed circuit board [1], railway [2], plastics [3], steel [4,5], glass bottle bottom [6], solar cell [7,8], fabric [9], and planar products [10]. However, most of these systems only detect the defects on the flat surface and are not useful for ski goggles lenses with a spherical surface. 

Visual defect inspection plays an important role in the ski goggles lenses manufacturing process. However, Taiwan’s ski goggles lenses industry currently lacks an efficient and low-cost defect inspection system. Detection and classification are implemented and recorded manually. The defect images are usually identified by experienced engineers or operators. This visual judgment method has some drawbacks such as being time-consuming, unconformity, and human fatigue, and then leads to misrecognition. In contrast, machine vision-based techniques have the advantage of high efficiency, low cost, objectivity, and so on, and are being widely applied to industrial defect inspection. Depending on the materials and surface shapes of the product, we have to design different image acquisition systems and therefore devise different strategies for defect detection and classification.

**Figure 2:**
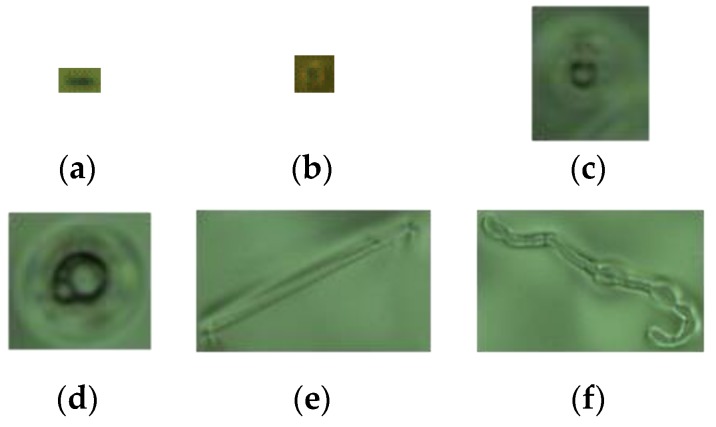
The dust and defect sample images. (**a**) Dust, sized 0.34 mm × 0.2 mm; (**b**) spotlight (type 1), size: 0.32 mm × 0.3 mm; (**c**) spotlight (type 2), size: 0.94 mm × 1.08 mm; (**d**) spotlight (type 3), size: 1.14 mm × 1.1 mm; (**e**) string, size: 2.56 mm × 1.54 mm; and (**f**) watermark, size: 3.62 mm × 2.56 mm.

To design an effective defect detection and classification system on ski goggles lens, we need to overcome some difficulties, as follows:

1. The ski goggles lenses have a spherical surface different in size and curvature, as shown in Figure 1. Thus, capturing a two-dimensional image of the entire ski goggles lenses is a major challenge;
2. Lenses are normally made from a polycarbonate or Trivex plastic. However, lenses are coated for multiple purposes with tints, anti-UV, or polarization, which have different light reflections. So, designing a lighting source for an image acquisition system that satisfies all types of ski goggles lenses is also a challenge;
3. High-quality ski goggles lens images mean high-resolution and larger sizes, so the algorithms for defect detection and classification need efficiency to reduce time in the production chain;
4. The defect detection and classification system not only assesses the quality of the product but also enables fast and accurate inspection of detecting faults early in the production chain. Therefore, the manufacturer is required to detect all types of defects with the size as small as possible, even very tiny as dust, which is very difficult to detect by a human. Besides, the defects have a similar shape, such as the dust and spotlight or the watermark and string, as shown in Figure 2.

To overcome the above challenges, we propose a novel framework based on machine vision, named ski goggles lens defect detection and classification system for real-time inspection and classification. First, we design an image acquisition system with high-resolution cameras and custom-made lighting field to get a high-quality lens image. Next, the defects on the ski goggles lens are detected by adopting parallel projection in opposite directions (PPOD) based on adaptive energy analysis. The PPOD method is used to detect the maximum and minimum value coordinates of defects based on the user’s requirement. Finally, the defect images and dust are classified into six types as dust, spotlight type 1, spotlight type 2, spotlight type 3, string, and watermark by the proposed classification algorithm. The defect detection and classification results of the ski goggles lenses are compared with the standard manufacturing quality. The experiments, which used ski goggles lenses provided by a company from Taiwan, demonstrate that the proposed method is sound and useful for ski goggles lens inspection. Not only does it reduce the number of human inspectors, but the yield rate can also increase, and material loss is reduced.

The remainder of this paper is organized as follows. Section 2 describes the image acquisition system. Section 3 presents the proposed defect detection method. Section 4 describes the classification algorithms. Section 5 discusses the experimental results. Finally, Section 6 gives the conclusions of this study.

## 2. Image Acquisition System

In this sub-section, the system architecture of the ski goggles lens (Foresight Optical Co., Ltd., Taiwan) inspection apparatus is first introduced, and particular considerations regarding the illumination scheme are presented. Then, the image properties and the challenges for defect detection are investigated.

In each image acquisition device, the lighting setup plays a major role in the quality of the acquired images, which is related to the success or failure of the inspection. When positive light meets an object surface, based on surface object material with different refractive indices, and depending on the angle of incidence and where the medium the light is coming from (e.g., air), some of the lights get reflected. On the other hand, the superfluous lights passing through the object reflect not only on the front surface but also on the backside, while the light may be reflected back and forth several times, leading to a captured image with varied quality. Therefore, the design of a suitable image acquisition system for each type of object plays a crucial role in the AOI system. This section introduces our image acquisition system, including light source setting, camera parameters setting, image capturing design, and so on.

**Figure 3:**
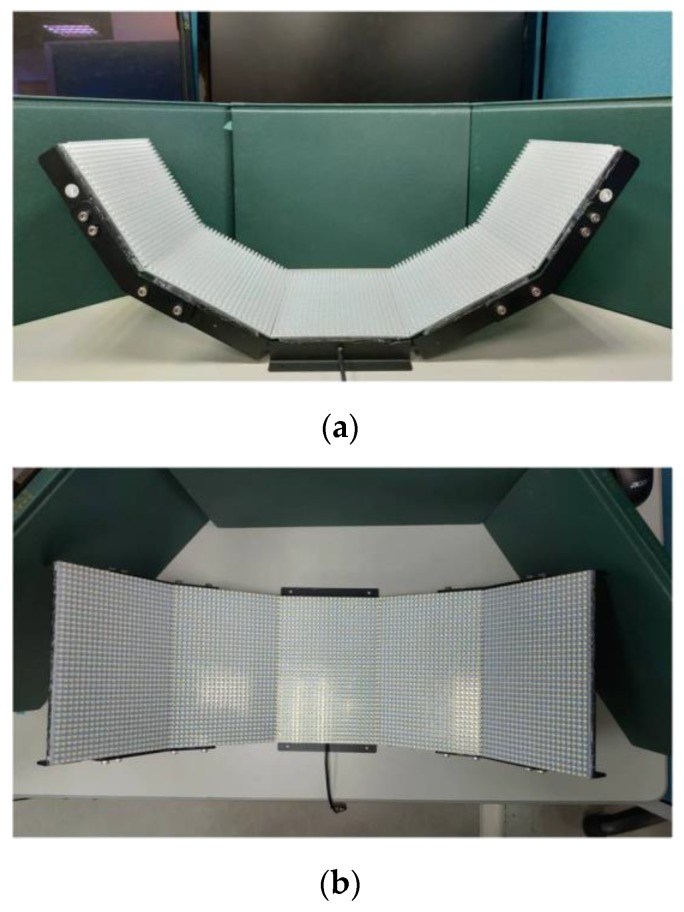
Lighting source pictures: (**a**) horizontally, (**b**) vertically.

To normalize illumination variation and increase the contrast between defect and background, lighting systems have been considered in this work, such as background lighting [11], parallel lighting [12], or infrared lighting [13]. Before choosing a suitable light source, it is necessary to prioritize the influence of the light source. The ski goggles lenses were made from a polycarbonate or Trivex plastic, which was coated for multi-purposes to protect the human eyes. They have a spherical surface different in size and curvature. Therefore, background lighting is used here. To create a homogeneous light field for the entire spherical surface of the lens, we designed a light source by using five pieces of flat light with surface-mounted LEDs, as shown in Figure 3. The flat light illuminated diffused light evenly at high output and helped the designed light source create a homogeneous light field for the entire spherical surface of the lens.

**Figure 4:**
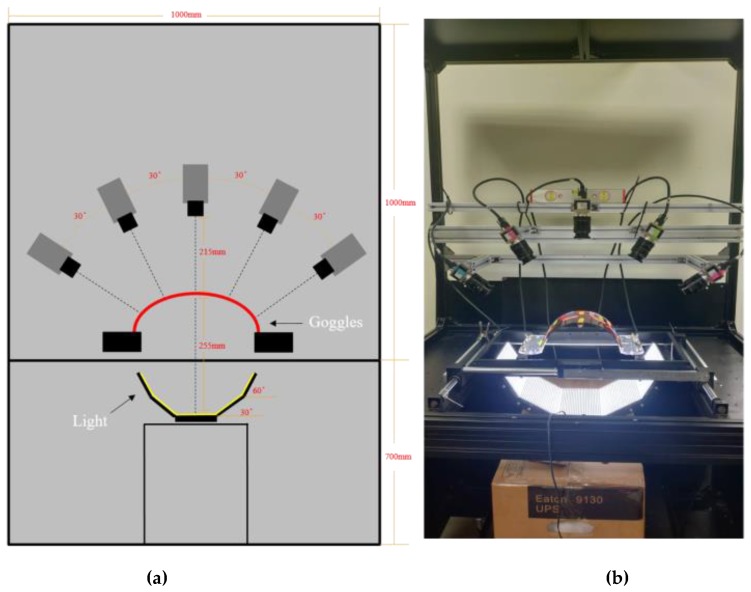
Diagram of our image acquisition system: (**a**) parameter setting of the system, and (**b**) real system image.

Next, we designed the camera system. Besides the light source setting, the distances among light sources, the camera with a lens, and test samples were examined to design an image acquisition system. For our experiment, symmetrical with five pieces of flat light, we attached five cameras. Each camera focused on part of the lens, which was illuminated by the corresponding flat light. Detailed parameters of the system are shown in Figure 4a and the real system image is shown in Figure 4b. The detailed hardware parameters are shown in Table 1.

**Figure 5:**
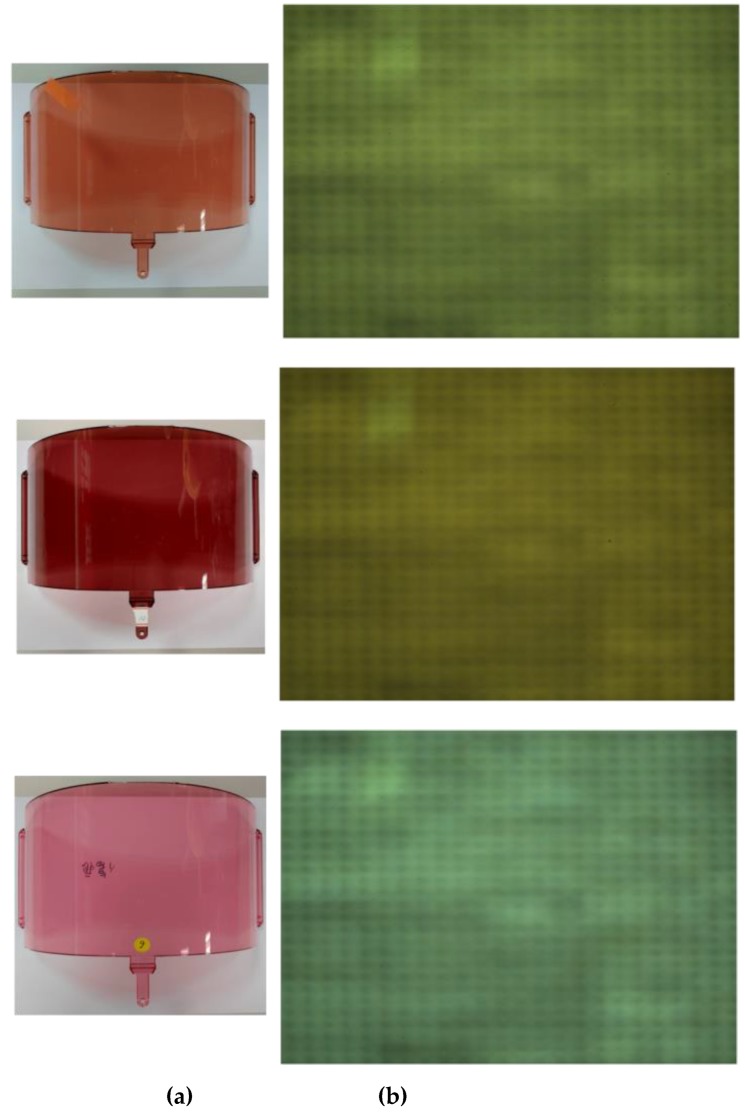
The example images of ski goggles lenses taken by our image acquisition system. (**a**) Ski goggles lens, and (**b**) the captured images.

In general, the defects on the ski goggles lens are small and very difficult to catch by human eyes. Therefore, image acquisition is one of the critical steps in the defect inspection system. Besides, to make the defect clearer, we designed an image acquisition system to take a high-resolution image. Three image examples captured by our system are shown in Figure 5, which demonstrates the ski goggles lens images to be examined in this study, measuring 4096 × 3000 pixels with a 24-bit BMP format. Table 2 shows the comparison between the ski goggles lens sample and its image was taken by our system. These images were tested not only for high defect detection rate but also minimized to decrease the running time, ensuring synchronization with the factory’s production line.

## 3. Ski Goggles Lens Defect Detection

This section introduces the ski goggles lens defect detection algorithm. To extract the correct position of the defect in the image, we detect the contour of the defect by following the defect shape. First, the adaptive energy analysis is used to enhance the defect contour, and then the PPOD method is used to cut out the defect image. The number and size of the defect are finally calculated and classified to determine whether the manufacturer’s specifications are met.

Traditional contour detection is based on edge detection approaches [14], e.g., Sobel or Canny operator, and commonly extracts edges by adopting a specific template or combining smooth function. However, the Sobel detector has a major drawback of being very sensitive to noise, and the performance of the Canny algorithm depends heavily on adjustable parameters, including the standard deviation for the Gaussian filter and threshold value. Researchers have recently developed an increasing number of edge detection operators, and each were designed to be sensitive to certain types of edges [15,16]. Unfortunately, these approaches usually require parameter tuning to adjust sensitivity throughout the image depending on contrast and differences. In this paper, the adaptive energy-based edge detector—which can not only detect edge pixels in all directions equally well but also has the advantages of no parameter tuning, low sensitivity to noise, and isotropy—is proposed.

Color is an effective and robust visual cue for distinguishing an object from others. Recently, there has been growing interest in color segmentation, which is useful in preliminary processing for many vision-based tasks, including object recognition, visual tracking, vision-based robotics, and more. However, contour detection is a technique for detecting meaningful discontinuities in the gray level and is often used in subsequent image analysis for feature extraction and object recognition. 

In digital image processing, CIELAB color space [17] has high stability in the multi-color mode, which can retain more complete energy information. Based on the usefulness of CIELAB color space, which describes the most complete color model visible to human eyes, we first convert the color defect image from RGB color space to CIELAB color space and then extract the $L^{*}$ channel containing more brightness information as a gray level for contour detection. It is not only more useful for defect detection but can also significantly reduce processing time. Instead of processing the 24-bit ski goggles lens color image, we processed the $L^{*}$ channel image of CIELAB color space with only 8 bits, thus saving the running time of the proposed system. 

**Figure 6:**
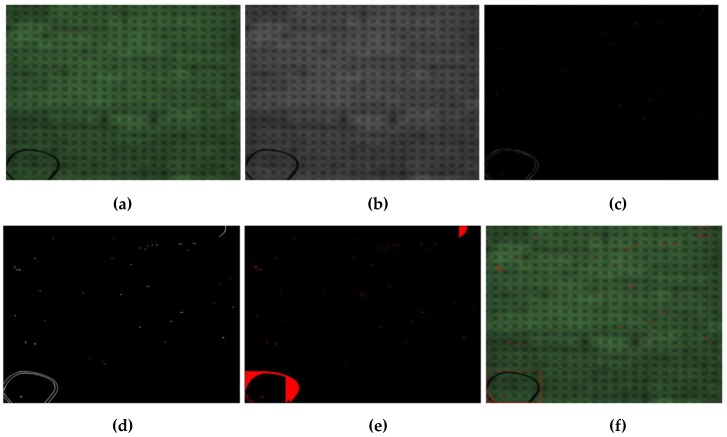
(**a**) Original ski goggles lens image; (**b**) $L^{*}$ channel in CIELAB image; (**c**) adaptive energy analysis image; (**d**) binary image obtained with Otsu’s method; (**e**) result of the parallel projection in opposite directions (PPOD) method; (**f**) result of defect detection.

Without loss of generality, f is assumed to be a ski goggles lens color image with a resolution of M × N, $f_{A},\;A\in \left\{R,G,\;B\right\}$ representing the RGB color channels; therefore, $f\in {ℜ}^{M\times N\times 3}$ and $\left\{f_{R},\;f_{G},f_{B}\right\}\in {ℜ}^{M\times N}$. The CIELAB color space is derived from the prior master CIE 1931 XYZ color space, which predicts which spectral power distributions will be perceived as the same color, but is not particularly perceptually uniform. To convert from RGB color space to CIE 1931 XYZ color space, the Equation

(1) $$\left[\begin{array}{c} X\\ Y\\ Z \end{array}\right]=\left[\begin{array}{ccc} 0.412453 & 0.357580 & 0.180423\\ 0.212671 & 0.715160 & 0.072169\\ 0.019334 & 0.119193 & 0.950227 \end{array}\right]\left[\begin{array}{c} R\\ G\\ B \end{array}\right],$$

and the parameters of CIELAB color space are calculated as:

(2) $$L^{*}\left(x,y\right)=116\times h\left(\frac{Y}{Y_{n}}\right)-16,$$

(3) $$a^{*}\left(x,y\right)=500\left[h\left(\frac{X}{X_{n}}\right)-h\left(\frac{Y}{Y_{n}}\right)\right]\;,$$

(4) $$b^{*}\left(x,y\right)=200\left[h\left(\frac{Y}{Y_{n}}\right)-h\left(\frac{Z}{Z_{n}}\right)\right]\;,$$

where

(5) $$h\left(q\right)=\{\begin{array}{cc} \sqrt[{3}]{q}, & \;\;q>0.008856\\ 7.787q+\frac{16}{116}, & otherwise \end{array}\;,$$

and $L^{*}$ for the lightness from black (0) to white (100), $a^{*}$ from green (−) to red (+), and $b^{*}$ from blue (−) to yellow (+). CIELAB was designed so that the same amount of numerical change in these values corresponds to roughly the same amount of visually perceiving change. *X_n_*, *Y_n_*, and *Z_n_* are the CIE XYZ tristimulus values of the reference white point (the subscript n suggests “normalized”). The $L^{*}$ channel image in CIELAB color space is shown in Figure 6b.

Next, for object contour extraction, an adaptive energy-based edge detector is used to smooth the $L^{*}$ channel image and enhance the desired defect edge. The energy *e* is defined by the mask with 3 × 3 pixels are presented in the following expressions:

(6) $$e\left(x,y\right)=\;\frac{1}{\eta }\overset{1}{\underset{i=-1}{\sum }}\overset{1}{\underset{j=-1}{\sum }}{\left\{L^{*}\left(x+i,y+j\right)–\mu_{L^{*}}\right\}}^{2},$$

where

(7) $$\mu_{L^{*}}=\frac{1}{\eta }\overset{1}{\underset{i=-1}{\sum }}\overset{1}{\underset{j=-1}{\sum }}L^{*}\left(x+i,y+j\right)$$

is the local standard average value of the pixels in the mask and η=9 is a normalizing constant.

To enhance the object of interest from the background, the binary image based on an automatic threshold proposed by Otsu [18] is adopted. The Otsu algorithm for the automatic binary threshold $\tau_{Otsu}$ is as follows:

(8) $$\tau_{Otsu}=max\left(\omega_{1}\left(t\right)\omega_{2}\left(t\right){\left[\mu_{1}\left(t\right)-\mu_{2}\left(t\right)\right]}^{2}\right),$$

(9) $$\omega_{1}\left(t\right)=\overset{t-1}{\underset{i=0}{\sum }}p\left(i\right),$$

(10) $$\omega_{2}\left(t\right)=\overset{255}{\underset{i=t}{\sum }}p\left(i\right),$$

(11) $$\mu_{1}\left(t\right)=\frac{\sum_{i=t}^{255}p\left(i\right)*i}{\omega_{1}\left(t\right)},$$

(12) $$\mu_{2}\left(t\right)=\frac{\sum_{i=t}^{255}p\left(i\right)*i}{\omega_{2}\left(t\right)},$$

where *t* is the current histogram level value from 0 to 255; $\omega_{1}\left(t\right)$ is the cumulative probability from 0 to *t*−1; $\omega_{2}\left(t\right)$ is the cumulative probability of t to 255; $\mu_{1}\left(t\right)$ is the cumulative expected average of 0 to *t*−1; $\mu_{2}\left(t\right)$ is the cumulative expected average of *t* to 255; and *p*(i) is the probability of distribution in the image. The binary image based on an automatic threshold value $\tau_{Otsu},$ denoted as *B*, is selected from the energy e as following:

(13) $$B\left(x,y\right)=\{\begin{array}{c} 255,\;\;e\left(x,y\right)\geq \tau_{Otsu}\\ 0,\;\;otherwise \end{array},$$

where pixel values labeled 255 are objects of interest, and pixel values labeled 0 are undesired ones. The binary image is shown in Figure 6c.

To fix the exact location of the defect in the ski goggles lens, this study adopts the PPOD method to determine the detection range. The PPOD method includes two projection mirrors used interchangeably to detect marginal points on the contours of defects on the ski goggles lens image. For binary image *B*, the first projection, called the forward projection and denoted as $m_{F}$, runs from the top left to the bottom right of the image to obtain the maximum value coordinate in the projection. The second projection, called reverse projection, with the mask denoted as $m_{I}$, runs from the bottom right point back to the first left point of the image to obtain the minimum value coordinate in the projection. The mask $m_{F}$ is defined in Equation (14), while the mask $m_{I}$ is defined in Equation (15).

(14) $$m_{F}=\left[\begin{array}{ccc} B\left(x,y\right) & B\left(x+1,y\right) & B\left(x+2,y\right)\\ B\left(x,y+1\right) & B\left(x+1,y+1\right) & \;0\\ B\left(x,y+2\right) & \;0 & \;0 \end{array}\right]\;,$$

(15) $$m_{I}=\left[\begin{array}{ccc} \;0 & \;0 & B\left(x,y-2\right)\\ \;0 & B\left(m-1,n-1\right) & B\left(x,y-1\right)\\ B\left(x-2,y\right) & B\left(x-1,y\right) & B\left(x,y\right) \end{array}\right]\;.$$

**Figure 7:**
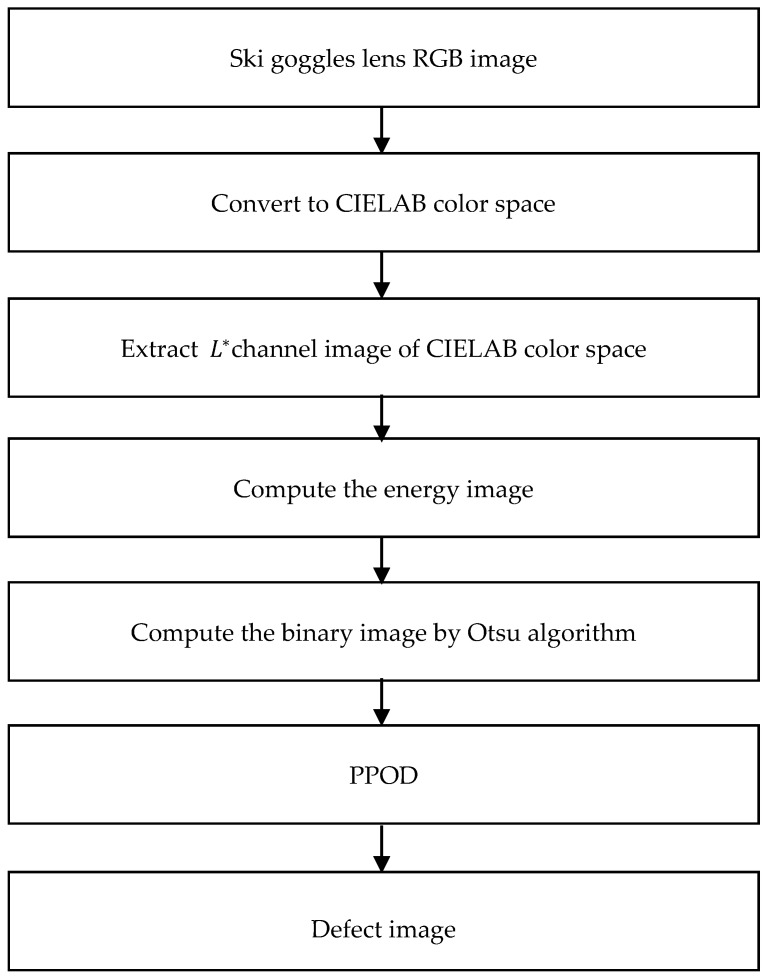
The defect detection algorithm diagram.

Based on the result of PPOD, the defects are automatically extracted to take down their numbers, sizes, coordinates, and classification. The manufacturer’s specification is then used to determine the defects of whether to meet the manufacturer’s specifications. Figure 6 shows the overall result of our defect detection method. Figure 6a shows the original ski goggles lens taken by our acquisition image system. Figure 6b shows the channel $L^{*\;}$ of the original image in CIELAB color space. Figure 6c shows the adaptive energy image of Figure 6b. Figure 6d shows the results of the binary image by Otsu’s algorithm, Figure 6e shows our PPOD method and Figure 6f shows the defect detection result. The overall defect detection algorithm diagram is shown in Figure 7. As the results can be seen, the defects are detected accurately.

## 4. Defect Classification

In this section, we propose the algorithms to classify the defects into four main types such as dust, spotlight, string, and watermark, while the spotlights will be classified into three types, named spotlight type 1, spotlight type 2, and spotlight type 3, as shown in Figure 2. This is the requirement from the manufacturer because our defect detection and classification system not only inspects the quality of the product but also enables fast and accurate inspection of detecting faults early in the production chain. Therefore, the manufacturer required the detection all types of defects with the size as small as possible, even very tiny as dust, which is very difficult to detect by a human. Although we designed an adaptive image acquisition system, however, due to the focal length problem caused by the curvature of the goggles, the intensity of the brightness, the weakening of the bulb, the different types of coated lens, etc., lead to the image obtained being uneven in brightness. The front and back scenes were not obvious enough, as shown in Figure 2. Therefore, the image of the defect is enhanced by an effective method before classifying.

### 4.1. Defect Image Enhancement

**Figure 8:**
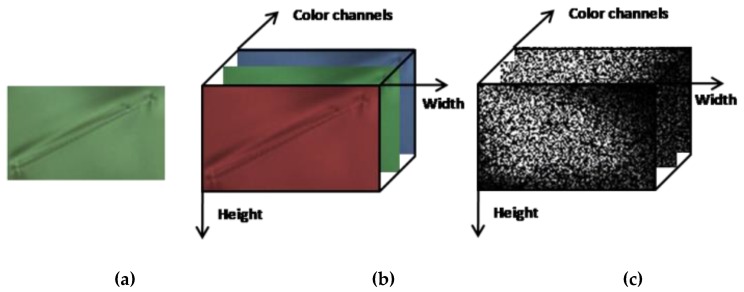
(**a**) A color defect image (**b**) illustrated as a third-order tensor. (**c**) The core tensor of (b) by applying high-order singular value decomposition (HOSVD).

For color image enhancement, many researchers have tried to reduce the effect of illumination on the image. Recently, some researchers were successful in applying singular value decomposition (SVD) for color face image enhancement [19,20,21,22,23]. However, these methods are useful for natural light sources, and not suitable for ski goggles defect color images taken under custom light sources. In this sub-section, we present a color defect image as a third-order tensor and propose a method to enhance the defect image by analysis the component of the tensor. By using high-order singular value decomposition (HOSVD) to decompose the defect color image of Figure 8a, we can find the interdependence of the structural components of the two dimension (2D) digital image.

Over the past two decades, the use of tensors and their decomposition has become increasingly popular. In multilinear algebra, HOSVD of a tensor is a specific orthogonal Tucker decomposition [24,25,26]. The power of a tensor framework can be presented in a visually and mathematically compelling manner by decomposing and representing an image in terms of its causal factors concerning data formation. HOSVD has been successfully applied to signal processing as well as big data [27], computer vision [28], and facial recognition [29].

The tensor is defined as a multidimensional array [30]. More formally, an N-way or Nth-order tensor is an element of the tensor product of N vector spaces, each of which has its coordinate system. For instance, an RGB color defect image of size M×N is expressed as a third-order $A\in {\mathbb{R}}^{M\times N\times 3}$ space, where *M* and *N* correspond to the x- and y-resolutions, respectively, and the number of color channels is three, as shown in Figure 8b. The tensor A includes three frontal slices denoted by Ai (the first, second, and third frontal slices are R, G, and B color channels, respectively). The HOSVD of tensor A can be expressed as

(16) $$A\;=\;S\times_{1}U_{1}\times_{2}U_{2}\times_{3}U_{3},$$

where $U_{i},\;i=1,2,3$ are matrices and called the inverse factors of A, and S is the core of tensor A. Given tensor A, the core tensor S is computed as:

(17) $$S=A\times_{1}U_{1}^{T}\times_{2}U_{2}^{T}\times_{3}U_{3}^{T},$$

The core tensor  S and its frontal slices are presented in Figure 8c.

**Figure 9:**
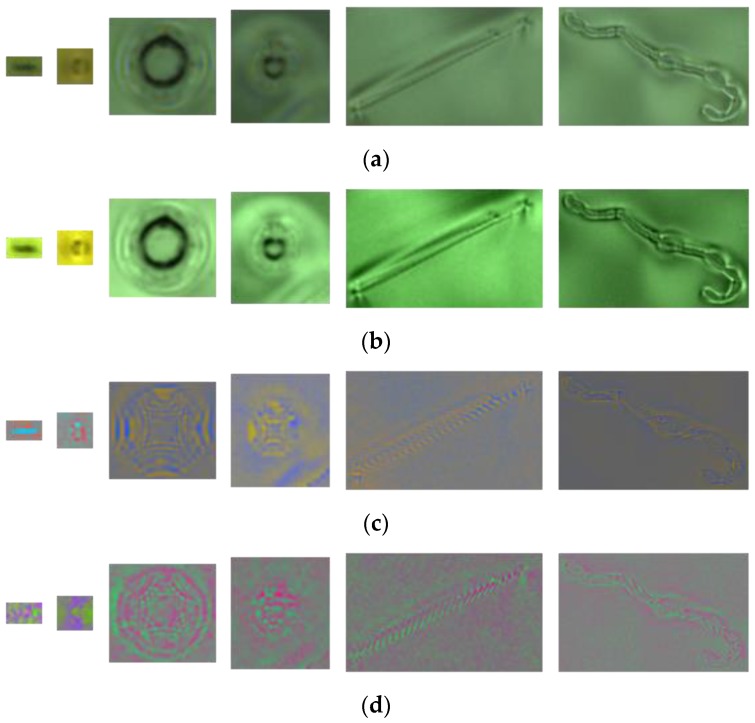
Effect of the core tensor presented on six defect images. (**a**) Six color defect images; (**b**) the reconstructed defect images of (**a**) by keeping the first frontal slice only; (**c**) the reconstructed defect images of (**a**) by keeping the second frontal slice only;and (**d**) the reconstructed defect images of (**a**) by keeping the third frontal slice only.

Unlike SVD, the core coefficients are not only located on the main diagonal of the frontal slices and evenly distributed across the frontal slices, but large values are also distributed around the main diagonal lines. To analyze in more detail the effect of the core tensor on the appearance of color defect images, by turn for using only one of the three frontal slices of core S  and assigning the remaining frontal slices to **O** matrices. **O** is an all-zero matrix, where every element is equal to zero. Figure 9a presents six color defect images of ski goggles lenses. Figure 9b shows the reconstructed images of Figure 9a by keeping the first frontal slice of core S unchanged and assigning the other two frontal slices to all-zero matrices. Figure 9c shows the reconstructed images by keeping the second frontal slice of core S and assigning the other two planes to O. Similarly, Figure 9d presents the reconstructed face images when the third frontal slice of core S is fixed and the other two planes are assigned to O. As seen, the first frontal slice of the core contains the most spatial structure (edge) of a defect. Besides, most of the reconstructed defect images, by keeping the first plane of core tensor and assigning the other two planes to O, look more clear. Therefore, we use them for classification.

### 4.2. Defect Image Classification

After extracting defects by the proposed algorithm, the defects are then used to classify based on the manufacturer’s specification. Based on our defect detection results, we can manually classify defects into five types, such as spotlight (type 1, type 2, and type 3), string, and watermark. The defects have different sizes and low contrast between the defect and background; even a tiny defect embedded in a ski goggles lens image may differ only slightly from the surrounding region. All these properties make the inspection extremely hard. This section proposes an effective method to classify defects in the ski goggles lens with a high accuracy rate.

#### 4.2.1. Classification of Dust and Defect

Dust is a physical substance produced by the environment. It will adhere to the surface of the ski goggles lens before coating, and the light will go through it. However, when the lens is coated, the dust is the cause of light refraction on the coated lens. To classify the dust and defects, we analyze the change of the grayscale value of the horizontal line going through the center of the defect.

**Figure 10:**
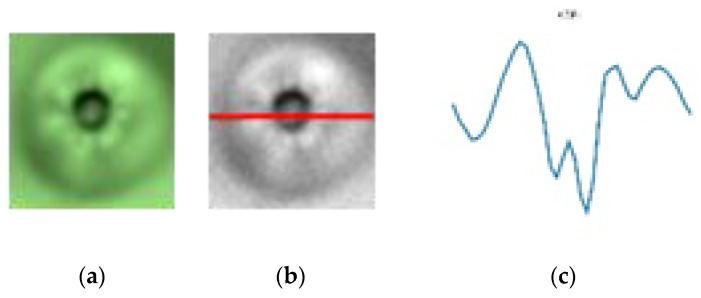
(**a**) The defect image; (**b**) the horizontal line going through the center of the defect; (**c**) the gray value variation along the horizontal line.

**Figure 11:**
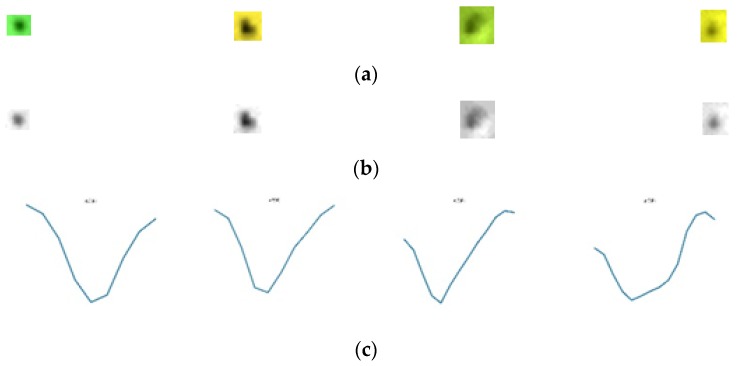
(**a**) The dust images; (**b**) the enhanced dust images by using HOSVD; (**c**) the variation of the gray level values.

**Figure 12:**
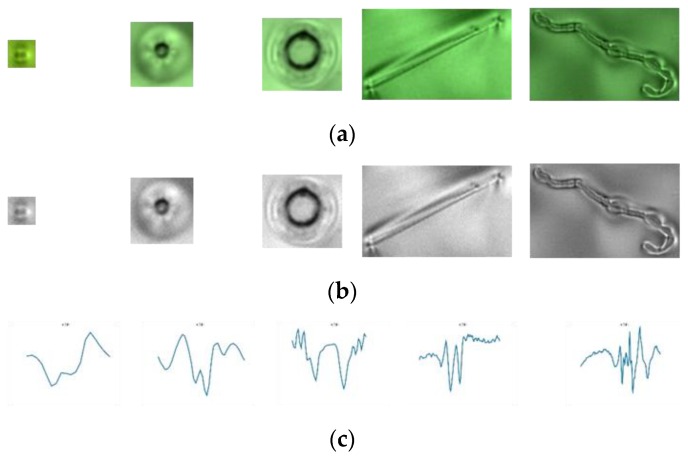
(**a**) The defect images; (**b**) the enhanced defect images by using HOSVD; (**c**) the variation of the gray level values.

First, the enhanced defect image is converted to a grayscale image and then has a horizontal line go through the center of the defect, as shown in Figure 10b. Later, the variation of gray level values of this line are computed. Without loss of generality, let *h*(*x*), where *x* = 1, 2, ... , *M*, be a function presenting the grayscale of the horizontal line going through the center of the defect image. The variation of the gray level of values, the horizontal line going through the center of the defect, is presented by the graph of the function *h*(*x*), as shown in Figure 10c. The variation of gray level values of the horizontal line going through the center of the defect images is shown in more detail in Figure 11 and Figure 12, respectively. As can be seen in the image, the variation of gray level values of the horizontal line going through the center of the dust images, as shown in Figure 11c, has a parabolic shape, which is very different when compared with the variation of gray level values of the horizontal line going through the center of the defect image, as shown in Figure 12c. Therefore, we classify the dust and defect by computing the number of extreme points of *h*(*x*) function.

Extreme points are places where a function takes on an extreme value that is especially small or especially large in comparison to other nearby values of the function. The *h*(*x*) function reaches extreme at a point $x_{k}$ if, and only if,

(18) $$h\left(x_{k}-1\right)\leq h\left(x_{k}\right)\;\mathrm{and}\;h\left(x_{k}+1\right)\leq h\left(x_{k}\right),$$

or

(19) $$h\left(k-1\right)\geq h\left(x_{k}\right)\;\mathrm{and}\;h\left(x_{k}+1\right)\geq h\left(x_{k}\right).$$

Denote $No._{ex}$ is a number of the extreme points of *h*(*x*) function, and an algorithm is designed to separate dust from defect as follows:

(20) $$\mathrm{Defect}\;\mathrm{image}=\{\begin{array}{c} \mathrm{dust},\;\;No._{ex}\leq 2\\ \mathrm{defect},\;\;otherwise \end{array}$$

#### 4.2.2. Classification of Point (Spotlight Types) and Line(String and Watermarking) 

In this sub-section, we propose a method based on the shape of a defect to classify the defects into two groups. Group 1 includes the spotlight defects that have point shape. Group 2 includes string and watermarking defects that have a line shape.

**Figure 13:**
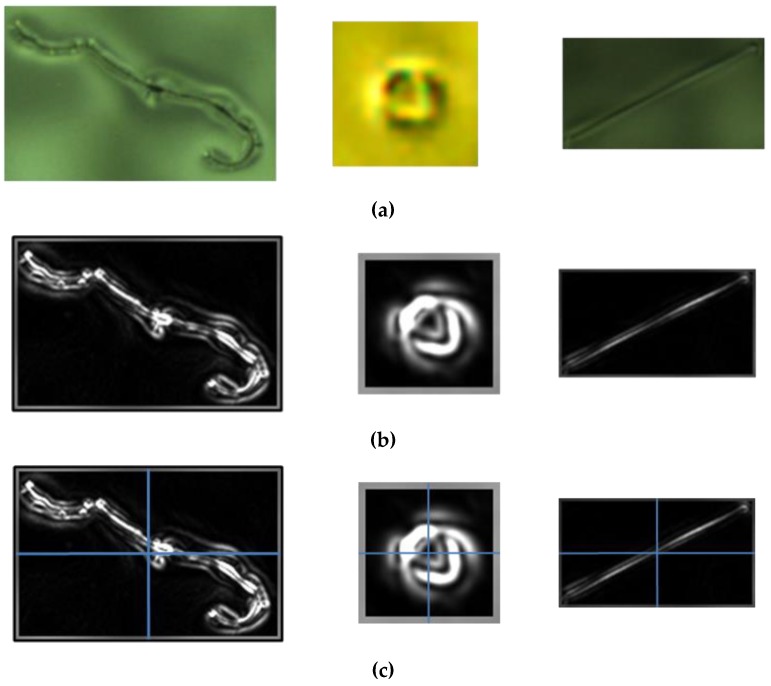
(**a**) Bright defect images; (**b**) defect region separation from the background, and (**c**) four equal parts of defect images.

First, we convert the enhanced color defect image f sized M×N from RGB color space to CIELAB color space and then extract the L* channel as a gray level for contour detection by adaptive energy-based edge detector as discussed in Section 3. Next, we separate the defect region from the background of the defect image as shown in Figure 13a. Here, we set the white color (grayscale value of 255) for the points within the defect region and black color (grayscale value of 0) for the background, as presented in Figure 13b. The image in this step is denoted as *B*. The *B* image is then divided into four equal parts along two axes through the center of the defect image and perpendicular to the edges, as shown in Figure 13c. The classification of string and point defects is done by calculating and comparing the ratio among the average of grayscale values of four equal parts. 

Then, we separate the defect region from the background of the defect image *f* sized M × N by using adaptive energy analysis, as discussed in Section 3. Here, we set the white color (grayscale value of 255) for the points within the defect region and black color (grayscale value of 0) for the background, as presented in Figure 10b. The image in this step is denoted as *B*. The image *B* is then divided into four equal parts along two axes through the center of the defect image and perpendicular to the edges, as shown in Figure 10c. The classification of string and point defects is done by calculating and comparing the ratio among the average of grayscale values of four equal parts, as follows:

(21) $$\mu_{B}=\frac{1}{M\times N}\overset{M-1}{\underset{x=0}{\sum }}\overset{N-1}{\underset{y=0}{\sum }}B\left(x,y\right)\;,$$

(22) $$\mu_{B1}=\frac{1}{\mu_{B}}\times \frac{4}{M\times N}\times \overset{M-1}{\underset{i=\frac{m}{2}}{\sum }}\overset{\frac{N}{2}-1}{\underset{y=0}{\sum }}B\left(x,y\right),$$

(23) $$\mu_{B2}=\frac{1}{\mu_{B}}\times \frac{4}{M\times N}\times \overset{\frac{M}{2}-1}{\underset{x=0}{\sum }}\overset{\frac{N}{2}-1}{\underset{y=0}{\sum }}B\left(x,y\right),$$

(24) $$\mu_{B3}=\frac{1}{\mu_{B}}\times \frac{4}{M\times N}\times \overset{\frac{M}{2}-1}{\underset{i=0}{\sum }}\overset{N-1}{\underset{j=\frac{N}{2}}{\sum }}B\left(x,y\right)\;,$$

(25) $$\mu_{B4}=\frac{1}{\mu_{B}}\times \frac{4}{M\times N}\times \overset{M-1}{\underset{i=\frac{M}{2}}{\sum }}\overset{N-1}{\underset{j=\frac{N}{2}}{\sum }}B\left(x,y\right)\;,$$

where $\;\;\mu_{B}$ is the average of the grayscale value of four quadrants, and $\;\mu_{Bi},\;i=1,2,3,4,\;\mathrm{is}$ the average percentage of the *i*-th quadrant referred to as $\mu_{B}$. Based on our observations, the defect region of Group 1 (point shape) is usually distributed into four parts of the defect image, while Group 2 (line shape) defects usually focus on one or two quadrants of the image. 

Denote sets *S*_1_ and *S*_2_ $\mathrm{with}\;\mu_{Bi},\;i=1,2,3,4$, that can be defined as follows:

(26) $$S_{1}=\{\mu_{Bi}>0.25,\;i=1,2,3,4\},$$

(27) $$S_{2}=\left\{\mu_{Bi}\leq 0.25,\;i=1,2,3,4\right\}.$$

Denote $\mu_{S1}$ and $\mu_{S2}$ as the average values of all elements in sets *S*_1_ and *S*_2__._ Let $n(S_{1}$) and $n(S_{2}$) as the element numbers of *S*_1_ and *S*_2.,_ while $\mu_{S1}\;\mathrm{and}\;\mu_{S2}\mathrm{can}\;\mathrm{be}$ calculated as follows:

(28) $$\mu_{S1}=\frac{1}{n\left(S_{1}\right)}\underset{\mu_{Bi}\in S_{1}}{\sum }\mu_{Bi},$$

(29) $$\mu_{S2}=\frac{1}{n\left(S_{2}\right)}\underset{\mu_{Bi}\in S_{2}}{\sum }\mu_{Bi}.$$

The classification of point and line defects is done as follows:

(30) $$\mathrm{defect}=\{\begin{array}{c} \mathrm{line},\;\mathrm{if}\;\{\begin{array}{c} n\left(S_{1}\right)\geq 2\\ s<\frac{1}{2}\mu_{S1},\forall s\in S_{1} \end{array}\\ \mathrm{point},\;\;\mathrm{otherwise} \end{array}$$

#### 4.2.3. Classification of Spotlight Defect into Three Types 

In this sub-section, we propose a method based on HOSVD and entropy to classify the point shape defects into three types of spotlight defects such as spotlight type 1, type 2, and type 3. First, type 1 of spotlight defects is classified from point shape defects, and the remains are classified into type 2 or type 3.

**Figure 14:**
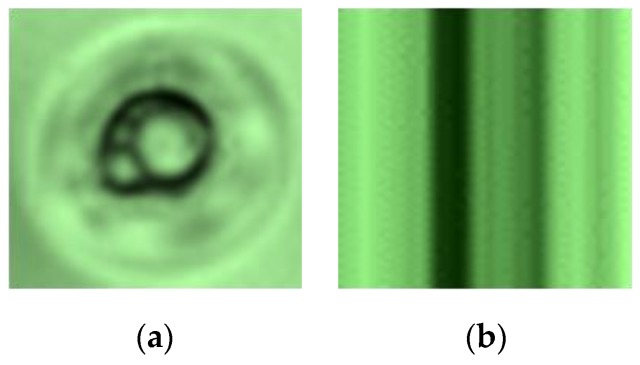
(**a**) A point shape defect image; (**b**) the reconstructed point shape image by replacing the first inverse factors U_1_ by an identity matrix **I**.

Here, we use the HOSVD to analyze the point shape defect image as shown in Figure 14a. The reconstructed point shape image is replaced with the first inverse factors U_1_ by an identity matrix **I** as shown in Figure 14b,

(31) $$A_{1}\;=\;S\times_{1}I\times_{2}U_{2}\times_{3}U_{3}.$$

**Figure 15:**
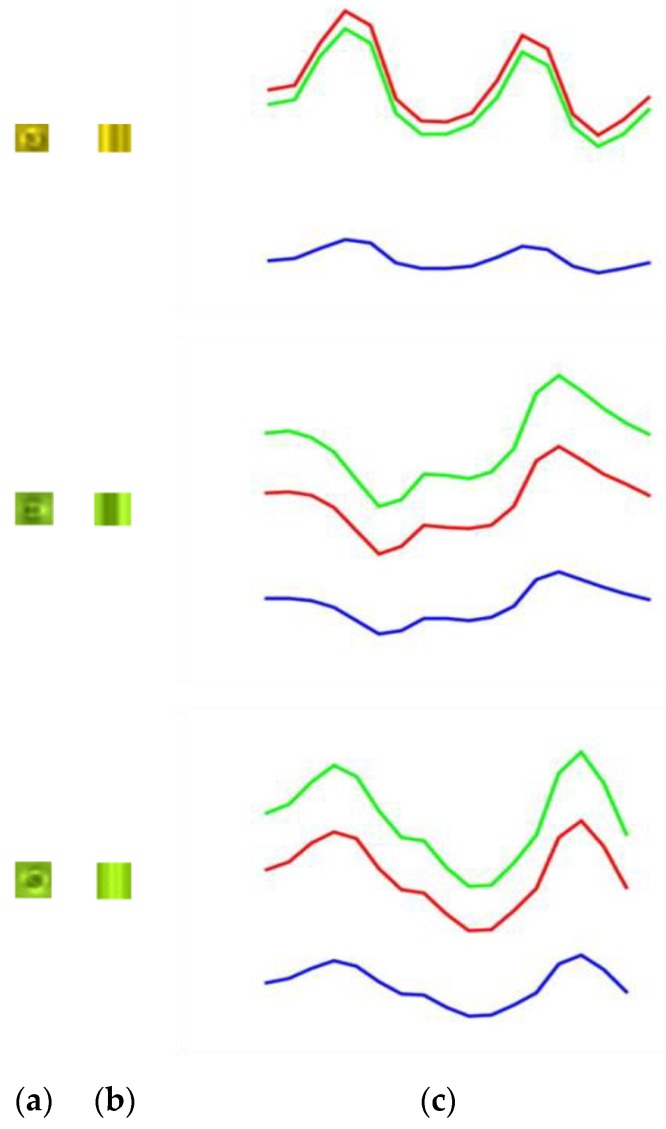
(**a**) Spotlight type 1 defect images; (**b**) the reconstructed images by replacing the first inverse factors U_1_ by an identity matrix **I**; (**c**) the distribution of three color channels of the spotlight defect images.

**Figure 16:**
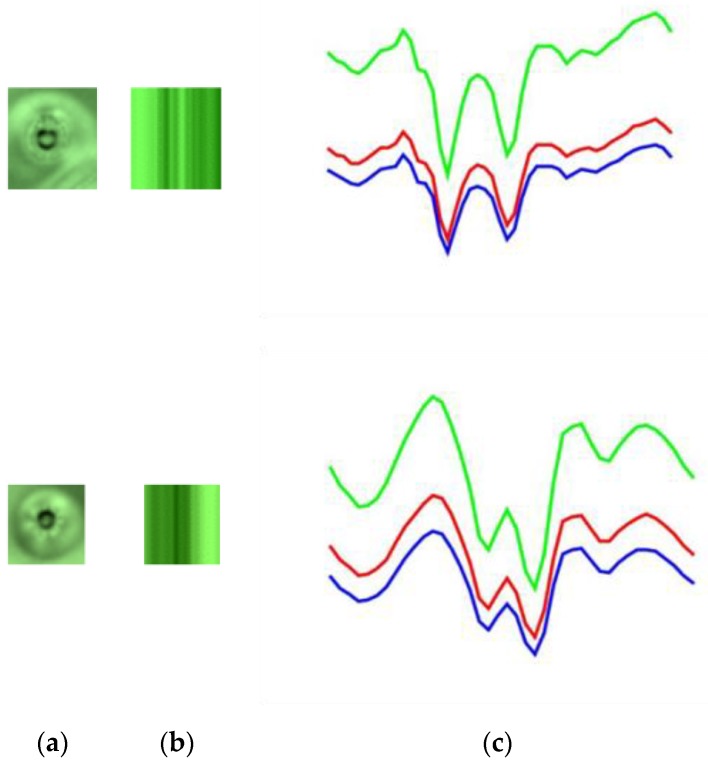
(**a**) Spotlight type 3 defect images; (**b**) the reconstructed images by replacing the first inverse factors *U*_1_ by an identity matrix **I**; (**c**) the distribution of three color channels of the spotlight defect images.

**Figure 17:**
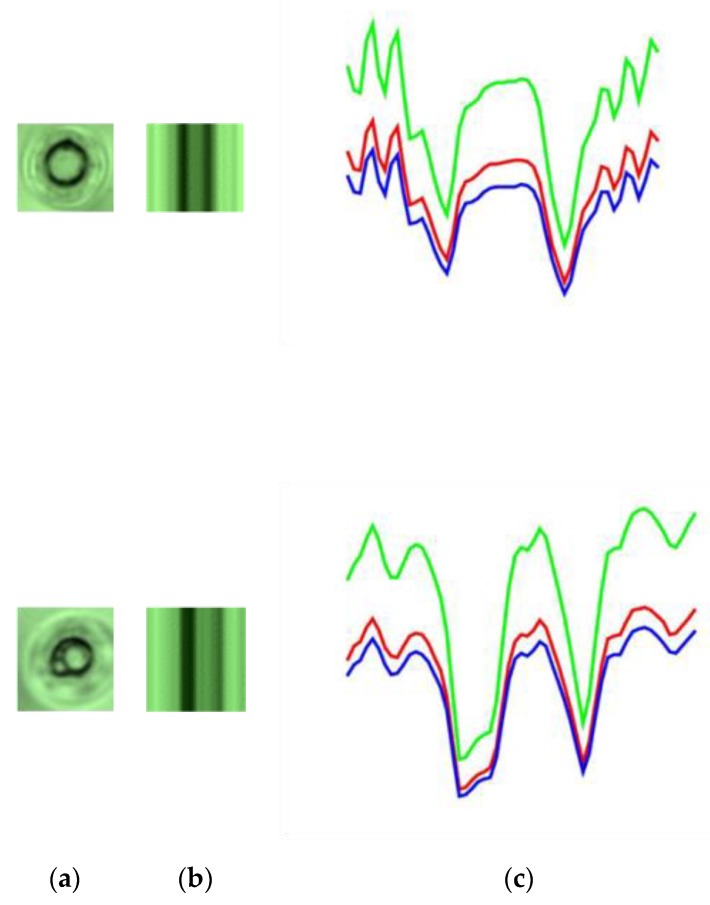
(**a**) Spotlight type 3 defect image; (**b**) the reconstructed image by replacing the first inverse factors *U*_1_ by an identity matrix **I**; (**c**) the distribution of three color channels of the spotlight defect image.

Based on our observation, the distribution of the three color channels of type 1 spotlight defect images is more different than the remaining spotlight types, as shown in Figure 15, Figure 16 and Figure 17. Therefore, type 1 spotlight defect is obtained by comparing the maximum, minimum, and median values of three color channels, as follows:

(32) $$m_{i}=\frac{1}{m\times n}\overset{m-1}{\underset{u=0}{\sum }}\overset{n-1}{\underset{v=0}{\sum }}A_{1}{}_{i}\left(u,v\right),\;i=\left\{R,\;G,\;B\right\}$$

(33) $$m_{a}=\;max_{i=R,\;G,\;B}(m_{i}),$$

(34) $$m_{b}=\;median_{i=R,\;G,\;B}(m_{i})$$

(35) $$m_{c}=\;min_{i=R,\;G,\;B}(m_{i})$$

(36) $$\;l_{1}=m_{a}-m_{c},$$

(37) $$l_{2}=m_{a}-m_{b},$$

(38) $$l_{3}=m_{b}-m_{c},$$

(39) $$\mathrm{spotlight}=\{\begin{array}{cc} \mathrm{type}\;1, & l_{1}>l_{3}>l_{2}\\ \mathrm{type}\;2,\;\mathrm{type}\;3, & \mathrm{otherwise} \end{array}$$

Next, we classify spotlight type 2 and type 3 by comparing the mean, denoted as $\mu_{s}$, standard deviation, denoted as $\sigma_{s}$, and entropy, denoted as $e_{s}$, based on the largest color channel of the defect image. In digital image processing, entropy is a measure of an image information content, which is interpreted as the average uncertainty of the information source. The entropy of an image can be used for measuring image visual aspects [31] or for gathering information to be used as parameters for adaptive histogram equalization [32]. Entropy is widely used for measuring the amount of information within an image. Higher entropy implies that an image contains more information. The entropy of information *e* was introduced by Shannon [33] in 1948, and it can be calculated by the following Equation:

(40) $$e_{s}=-\overset{255}{\underset{i=0}{\sum }}p_{i}\log_{2}p_{i}$$

where $\;p_{i}$ denotes the probability mass function of gray level *i*, and it is calculated as follows:

(41) $$p_{i}=\frac{\mathrm{No}.\;\mathrm{of}\;\mathrm{occurrences}\;\mathrm{of}\;\mathrm{intensity}\;\mathrm{levels}}{\mathrm{No}.\;\mathrm{of}\;\mathrm{intensity}\;\mathrm{levels}}.$$

the type 2 and type 3 of the remained point shape defects are classified by the Equation:

(42) $$\mathrm{spotlight}=\{\begin{array}{cc} \mathrm{type}\;3, & \mu_{s}-\sigma_{s}\leq e_{s}\leq \mu_{s}+\sigma_{s}\\ \mathrm{type}\;2, & otherwise \end{array}$$

#### 4.2.4. Classification of String and Watermark Defects

We make a comparison with mean *μ*_s_, standard deviation *σ*_s_, and entropy *e*_s_ of the largest color channel for the defect image to further classify lines into string and watermark, as follows:

(43) $$\mathrm{line}=\{\begin{array}{cc} \mathrm{string}, & \mu_{s}-2\sigma_{s}\leq e_{s}\leq \mu_{s}+2\sigma_{s}\\ \mathrm{watermark}, & otherwise \end{array}$$

**Figure 18:**
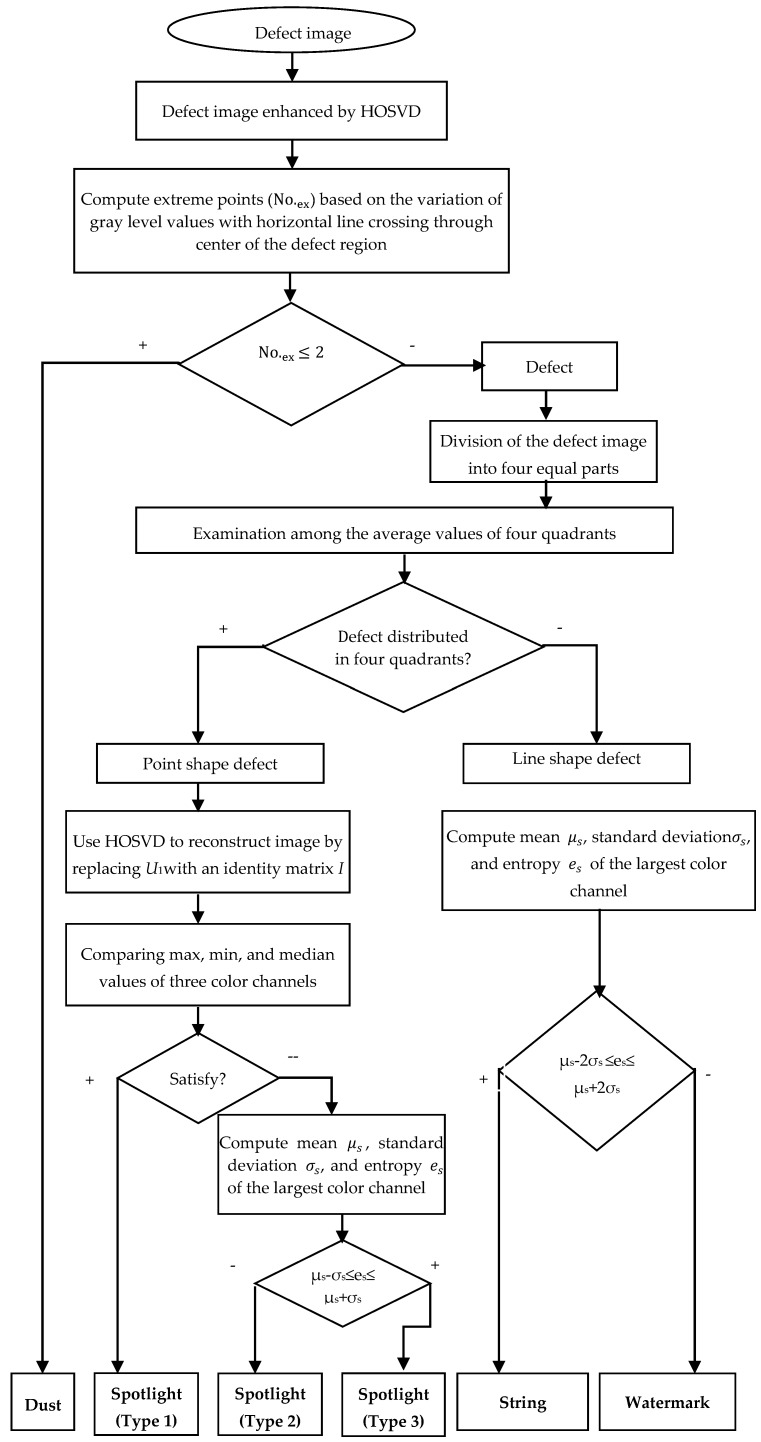
The classification algorithm diagram.

In summary, the defects have been classified into six types: dust, spotlight (type 1, type 2, and type 3), string, and watermark based on the manufacturer’s specifications. The overall classification algorithm diagram is shown in Figure 18.

## 5. Experiment Result and Discussion

In this section, the defect detection and classification algorithms are tested on four types of ski goggles lens samples with a total of 120 pieces provided by the cooperator, as shown in Figure 1.

### 5.1. Image Acquisition Setting

The proposed systems were implemented in Microsoft Visual Studio C++ 2010. The experiments were conducted on a PC with an Intel Core i7-4790 CPU @ 3.60 GHz, RAM DDR3 8 GB, running with Windows 10 operating system. The system used Basler avA2300-30kc camera with Camera-Link interface and equipped with KAI-4050 CCD sensor delivering 31 frames per second at 4 MP resolution, sensor size with 12.8 mm × 9.6 mm, resolution (H × V) with 4096 × 3000 pixels, pixel size (H × V) with 5.5 µm × 5.5 µm. The camera adapted was Nikon 60 mm f/2.8 microlens (Nikon, Minato, Tokyo, Japan). This lens is an excellent normal and short telephoto lens, as well as a superb close-up lens, which means an object as small as an inch across can fill the frame. The light source was equipped with PHILIPS Ambiance Globe 17W E27 Cool daylight (PHILIPS, Amsterdam, Netherlands).

### 5.2. Defect Detection Result

In order to inspect whether the ski goggles lens satisfies the requirement of the manufacturer or not, the ski goggles lens image was used to locate and extract the defects. The RGB image was transformed into CIELAB color space to get the L* channel image for contour extraction. Based on the localized energy analysis of the L* channel image, the image was converted to binary image based on the Otsu algorithm and then was used to located and extract the defects by the proposed PPOD method. The size and number of the defects were then calculated to check against the requirement of the manufacturer’s specification and later put to the classification process to make a decision whether it could pass or not. In our experiments, we achieved a 100% defect detection rate. The average time including load/unload for defect detection was 30 s/piece.

### 5.3. Defect Classification Result

Based on the manufacturer’s specifications, the defects were classified into six types: dust, spotlight (type 1, type 2, type 3), string, and watermark. The overall algorithm diagram is shown in Figure 18. First, the defect images were enhanced by HOSVD. Based on the analysis of the components in the core tensor, the most important information of defect was found in the reconstructed images by keeping the first plane of core tensor and assigning the other two planes to zero matrices. Therefore, the image of the defect was first enhanced by the proposed method before classifying it. Next, the dust image was classified from defects by computing the number of extreme points of variation of gray level values of a horizontal line going through the center of the defect. 

The defect image was then classified into two groups based on examination among the average values of four equal parts of the defect image. Group 1 included three types of spotlight defects with point shape, while Group 2 included the string and watermarking defects with line shape. For the spotlight defects, we first used HOSVD to reconstruct point shape images by replacing the first inverse factors U1 by an identity matrix I. To obtain type 1 of spotlight defects, we compared the value of the maximum, minimum, and median of three color channels of the reconstructed image. The type 2 and type 3 of the spotlight were then classified by comparing the mean, standard, and entropy of the strongest color channel of the defect image. For the line shape defect, we also computed mean, standard and entropy of the strongest color channel of the defect image to classify them into string defect and watermark. 

The experimental results show that our method is feasible in the ski goggles lens inspection system and more effective than the traditional human visual inspection method. Through experimental tests on 176 defects (classification rate of 98.6%) and 120 dust images (recognition rate of 100%), the presented method achieved a classification rate of 99.3% in accuracy and a running time of 0.5 s per sample in average. The results show that our system can detect and classify the defect in the ski goggles lens with high efficiency, even in case the size of the defect is very small and impossible to be seen clearly by human eyes. Moreover, the running time was short so the proposed framework could fully meet the manufacturer’s requirements. The proposed system not only inspects the quality of the product but also enables fast and accurate inspection of detecting faults in the early period of the production line.

### 5.4. In Comparison with Related Works

In general, researchers use modern statistical or data mining methods to resolve the classification problem [34]. However, the learning algorithms depend heavily on finding a good sparse feature vector, which is represented with an object [35,36,37,38]. Our method uses statistical features to distinguish various defect types. To demonstrate the generalizability of the proposed method, we did more experiments.

**Figure 19:**
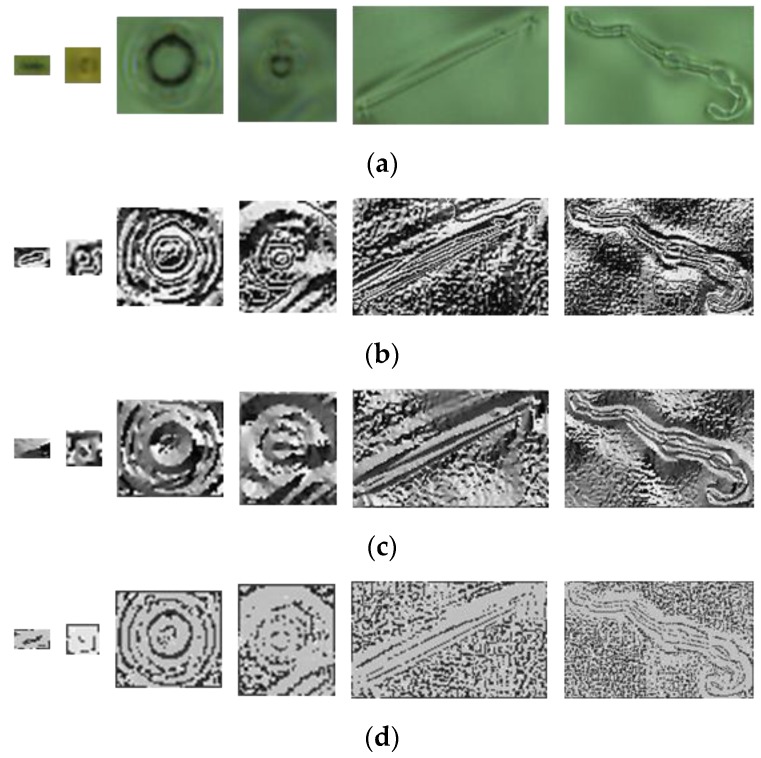
(**a**) The dust image (first image) and defect images (the other images); (**b**) result of applying the local binary pattern (LBP) method to (**a**); (**c**) result of applying the gradient method to (**a**); (**d**) result of applying the Weber method to (**a**).

We compared the defects enhanced by the proposed HOSVD method with other methods, such as local binary pattern (LBP) [39], gradient [40], and Webber’s law [40]. These methods are very useful to remove the effect of lighting on the object. Figure 19a presents original images with one dust image and five defect images,Figure 19b presents the result by applying the LBP method, Figure 19c presents the result by applying the gradient method, and Figure 19d presents the result with the Webber method. In our test, the most important task is to classify dust from defects. We computed the horizontal line going through the center of the defect and observed the variation of gray level values. 

**Figure 20:**
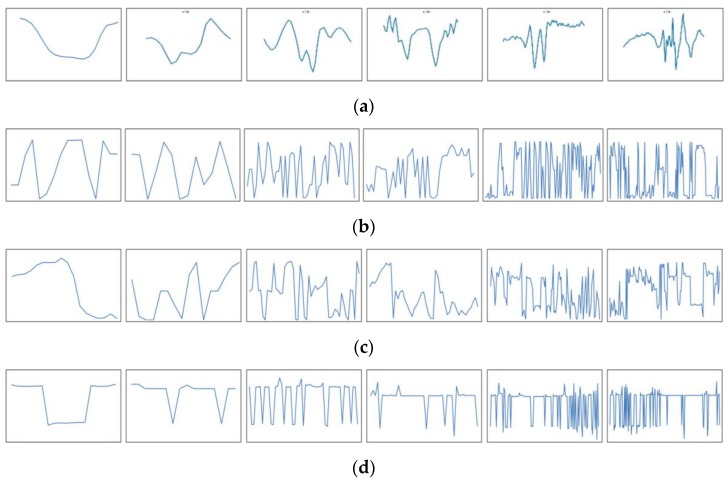
The variation of gray level values with the horizontal line going through the center of the enhanced defect images in Figure 19: (**a**) enhanced by the HOSVD method; (**b**) enhanced by the LBP method; (**c**) enhanced by the gradient method; and (**d**) enhanced by the Webber method.

Figure 20 presents the variation of gray level values in the horizontal line by going through the center of the defect image Figure 19. Figure 20 presents the variation of gray level values of the horizontal line going through the center of the dust images enhanced by the proposed HOSVD method. The first image in Figure 20a has a parabolic shape, which is very different when compared with the variation of gray level values of the horizontal line going through the center of the defect images. However, we cannot distinguish dust from defects when using LBP (Figure 20b), gradient (Figure 20c), and Webber (Figure 20d). Accordingly, by using a simple statistical feature obtained from the HOSVD image, we found it easy to classify dust from defects with a 100% true detection rate. On the other hand, we classified dust from defects by using a learning method with the support vector machine (SVM) [41]. We used the histogram of oriented gradients (HOG) descriptors [42] as a feature vector for SVM. In this study, the radial basis function kernel $\exp\left(-\gamma \|x-x^{\prime }{\|}^{2}\right)$, where x and **x′** are represented as feature vectors in input space, was used in SVM and the parameter ***γ*** is set to 20 with an experimental setting. However, the results were not as good as expected. We only got a 71% true classification rate. This was caused by the size of dust, in which the defect images were really different.

In this study, the optimized goggles detection system was used as the main axis, and the image capturing system was customer-designed to improve the performance of the goggles detection system. With the designated environment, taking the clear original image as the starting point, according to two major problems, the lighting source should uniformly light the whole goggles for capturing the original image, and therefore the shooting mode of five cameras is proposed. Based on the cameras and the angles of shooting, we can make each camera capture a smaller range, effectively avoiding classification errors caused by blurring with focus. In addition, the bowl shape LED light source was been customer-designed for the architecture of five cameras, the main purpose of which was to cover the entire goggles. According to the above experimental results, it is confirmed that the goggles detection system of this paper is optimized, which effectively increases the efficiency of the proposed system. The shooting structure of the five cameras can also accommodate more different goggles samples and obtain clear image matching and screening. The algorithm can greatly reduce the system operation time and is more suitable for production line use. The overall calculation time has been accelerated in this study so that the goggles detection system can achieve immediate operation.

## 6. Conclusions

In this study, we propose an effective ski goggles lens defect detection and classification system, which has three parts. The first part includes an optical structure for image acquisition that can remove natural light interference and then highlight defects. The second part includes an efficient algorithm called the PPOD method, which includes two interchangeable projection mirrors to detect marginal points on the contour of defect on the ski goggles lens image and then locate and extract the defect image. Finally, an efficient classification algorithm is proposed to classify the defects into six types: dust, spotlight (type 1, type 2, and type 3), string, and watermark based on manufactory requirements. The experimental results run on 120 ski goggles lens pieces provided by the cooperative manufacturer and have shown that our system can get a 100% defect detection rate and a 99.3% classification accuracy rate. Moreover, it only takes 0.5 s on average to perform the inspection for a piece of ski goggles lens.

## Figures and Tables

**Table 1:** The hardware parameters setting of our image acquisition system.

Item	Detail
Operating system	Windows 10 (64 bit) (Microsoft, Redmond, Washington, DC, USA)
CPU Info.	Intel (R) Core (TM) i7–4790CPU @ 3.60 GHz (Intel Corporation, Santa Clara, CA, USA)
Memory (RAM)	8 GB (Kingston Technology Company, Inc., Fountain Valley, CA, USA)
Capture card	EuresysGrablink Dual Base (Euresys, Liège, Belgium)
Camera	KAI-4050 CCD Sensor (ON Semiconductor, Phoenix, AZ, USA)
Optical lens	Tokina TC3520-12MP 35 mm F2.0–22 (Tokina, Nakano, Tokyo, Japan)
Light source	CCS TH-224X170SW (CCS Inc., Kyoto, Japan)

**Table 2:** The comparison between the ski goggles lens sample and its image.

Item	FOV	Inspection Scope	Resolution	Pixel Size
Profile	60 × 44 ${\mathrm{mm}}^{2}$	$2640{\;\mathrm{mm}}^{2}$	4096 × 3000 pixels	20 µm/pixel
